# Actions related to workers, employers, and the workplace associated with musculoskeletal and mental health diseases in workers on sick leave: a qualitative systematic review

**DOI:** 10.47626/1679-4435-2022-740

**Published:** 2023-02-13

**Authors:** María Cecilia Toffoletto, Jorge David Ahumada

**Affiliations:** 1 Enfermería, Universidad de las Américas, Santiago, Santiago, Chile; 2 Enfermería/Kinesiología, Inacap/ IPChile, Rancagua, Rancagua, Chile

**Keywords:** occupational diseases, return to work, mental disorders, musculoskeletal diseases, clinical trial, enfermedades profesionales, reinserción al trabajo, trastornos mentales, enfermedades musculoesqueléticas, ensayo clínico

## Abstract

The objective of this study was to describe the interventions for the labor reintegration of workers on medical leave due to musculoskeletal and mental health diseases, according to actions related to the worker, the employer, and the workplace. This study consists of a qualitative systematic review, without restriction of publication date, conducted in the Cochrane Central Register of Controlled Trials (CENTRAL) and MEDLINE/PubMed scientific bases. In addition, the Epistemonikos database was used. Nineteen articles were selected. It is observed that all interventions proposed actions with the workers, such as rehabilitation programs, therapies and return to work plans. Regarding the actions in the workplace, only three interventions articulated actions with workers and evaluation of the workplace. Finally, actions with employers were considered in 10 interventions with the objective of involving the employer in the improvement of the workplace and planning for the worker’s return to work. It can be seen that interventions for patients with musculoskeletal and mental health disorders can be divided into the following categories: worker-oriented interventions, employer-oriented interventions, and workplace actions. In each of these categories, various interventions can be seen, ranging from multidisciplinary intervention to exercise-based rehabilitation, in the case of musculoskeletal disorders, and occupational therapy to the psychotherapeutic method based on music, for mental health disorders.

## Abstract (es)

El objetivo de este estudio fue describir las intervenciones para la reinserción laboral de trabajadores en licencia médica por enfermedades musculoesqueléticas y de salud mental, en términos de acciones relativas al trabajador, al empleador y al lugar de trabajo. Este estudio consiste en una revisión sistemática cualitativa, sin restricción de fecha de publicación, en las bases científicas de Registro Cochrane Central de Ensayos Controlados (CENTRAL) y MEDLINE/PubMed. Además, se utilizó la base de datos Epistemonikos. Fueron seleccionados 19 artículos. Se observó que todas las intervenciones propusieron acciones con los trabajadores, tales como programas de rehabilitación, terapias y planes de regreso al trabajo. En cuanto a las acciones en el lugar de trabajo, solo tres intervenciones articularon acciones con el trabajador y evaluación del lugar de trabajo. Por último, las acciones con los empleadores fueron consideradas en 10 intervenciones, con el objetivo de involucrar al empleador en las mejorías del lugar de trabajo y planificación para el regreso al trabajo del trabajador. Se puede advertir que las intervenciones para los pacientes con trastornos musculoesqueléticos y de salud mental se pueden dividir en las siguientes categorías: intervenciones orientadas al trabajador, orientadas al empleador, y acciones en el lugar de trabajo. En cada una de estas categorías se pueden apreciar intervenciones variadas, que van desde la intervención multidisciplinaria hasta la rehabilitación en base a ejercicios, en el caso de los trastornos musculoesqueléticos, y de la terapia ocupacional hasta el método psicoterápico basado en la música, para los trastornos de salud mental.

## INTRODUCTION

The World Health Organization (WHO) reported the predominance of musculoskeletal and mental health disorders among work-related diseases in the last 15 years,^1^ which are responsible for work disability and a burden to society, workers, and organizations.^2^

Work disability refers to individuals who have discontinued their participation in occupational activities^3^ or to the result of a condition that causes a worker to miss at least one day of work and includes time off work, as well as any ongoing work limitations.^4^ There are three stages of disability, defined by the number of absent days: an acute stage (up to 1 month), a sub-acute stage (2-3 months), and a chronic stage (more than 3 months).^5,6^

With regard to musculoskeletal diseases, according to the Bureau of Labor Statistics of the United States Department of Labor, in 2002, 24.17% of a total of 347,000 work-related upper-limb injuries were work related should injuries.^7^ In Sweden, like in most Western countries, musculoskeletal disorders, especially those affecting neck, back, and shoulders, are one of the most common problems among retired people with an illness (> 90 days) and a disability.^8^

Concerning mental health disorders, are the most frequent of them are occupational stress, anxiety, depression, and burnout syndrome. A study that analyzed the relationship between anxiety and depression symptoms and socioeconomic level among technical-administrative employees of a public university in Brazil revealed a high prevalence of anxiety and depression among participants, with no relationship with their socioeconomic level. Stress was more frequent among participants with higher educational level.^9^

Among all issues related to occupational disorders, it is worth highlighting the significant economic impact of absenteeism, whose most prevalent causes worldwide are musculoskeletal and mental health disorders, with increased rates of stress, anxiety, depression, and even suicide.^10,11^

Therefore, considering the impact of lost days because of absenteeism, it is necessary to emphasize the importance of workplace reintegration focusing on relapse prevention and permanency in the job.^12,13^ Return to work after a sick leave due to an occupational disease is a complex and not always possible process.

In the literature, interventions aiming to reduce sick leave duration and facilitate return to work are very diverse. They are planned by health care providers and insurers and involve health care professionals such as physicians, occupational therapists, psychologists, among others. Furthermore, they consist of various activities, such as occupational therapy, kinesiology sessions, physical activity, psychological therapy, medical interventions, ergonomics in the workplace, and education, in addition to activities of problem solving in the workplace together with employers. Duration of interventions ranged from weeks to months or until worker’s full return to work.^14^

Conversely, despite abundant information in the literature based on studies addressing this issue, there is an important need to develop a systematic review to know what actions are proposed in interventions associated with musculoskeletal and mental health diseases in relation to workers, employers, and workplace, thus representing a useful bibliographic resource to discuss and propose occupational health policies that include this focus by area of intervention according to scientific evidence.

Based on the previously raised issue, the following question emerges: what actions with workers, with employers, and in the workplace are proposed in interventions for the labor reintegration of workers on sick leave for musculoskeletal and mental health diseases?

The present review aimed to describe the actions with workers, with employers, and in the workplace proposed in interventions for the work reintegration of employees on sick leave for musculoskeletal and mental health disorders.

## METHODS

This study is a qualitative systematic review, a type of research that aims to synthesize the produced knowledge on a specific theme and to present evidence in a descriptive manner, with no statistical analysis.

Inclusion criteria established for the articles were: randomized controlled trials (RCTs) that evaluated the effect of interventions for the labor reinsertion of workers with occupational diseases (musculoskeletal and mental health). Complete studies were included, without restriction of language or date of publication. Articles that compared return-to-work interventions with usual treatment were included. With regard to exclusion criteria established for articles, studies proposing a single intervention for different occupational diseases were not considered.

Interventions were defined as programs whose aim was to promote return to work with activities directed to factors related to the process of return to work. The outcomes analyzed in the articles included were time to full return to work, defined as the time elapsed from the start of sick leave until full return to work, measured in calendar days, weeks, and/or months, and proportion of workers who returned to work full time.

Systematic search was conducted in the Cochrane Central Register of Controlled Trials (CENTRAL) and MEDLINE/PubMed databases. Furthermore, the Epistemonikos database, which is able to screen 30 databases, was used to identify systematic reviews and the primary studies included in them. The descriptors used were “Clinical Trial” [Mesh] AND “Return to Work” [Mesh] AND “Occupational Diseases” [Mesh] AND “Musculoskeletal Diseases” [Mesh]) AND “Mental Disorders” [Mesh]. Furthermore, a decision was made to use the descriptor “Intervención” in the literature search.

In total, 4,046 scientific articles were identified. Of these, 2,878 were excluded after reading of their abstract, because they were not RCTs, and 1,129 because they did not meet inclusion criteria. The remaining 39 studies were independently read in full by each investigator.

**Figure 1:**
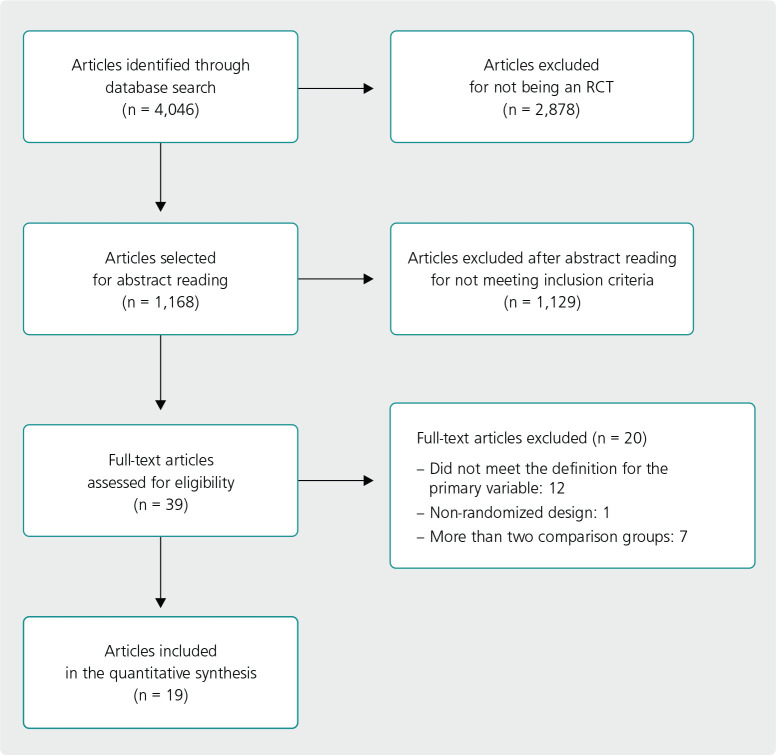
Flowchart: selection of articles for inclusion.

Twenty articles were excluded for the following reason: non-compliance with the definition for the primary result (12 articles), non-randomized design (1 article) and, finally, because they presenting more than two comparison groups (7 articles). Therefore, 19 articles were included in the present review for qualitative synthesis (Figure 1).

## RESULTS

Table 1 presents the interventions according to health problem and actions proposed in relation to workers, employers, and the workplace.

**Table 1:** Description of interventions according to disease and actions related to workers, employers, and the workplace

Intervention and authors	Disease	Actions with workers	Actions in the workplace	Actions with employers
Workplace ergonomic intervention. Arnetz et al.^15^	Musculo­skeletal	Training program included information on the type of training and work assignments adapted to employee’s ability, the time assigned for each training session, weeks of training, and a program for a successive increase in workload.	The workplace was assessed from the ergonomic point of view. The ergonomist evaluated the physical and psychosocial stressful factors while employees performed their usual work tasks. When appropriate, ergonomic improvements were introduced.	The employee, the insurance care manager, the occupational therapist/ergonomist, and the employer held a meeting at the workplace to introduce the ergonomic improvements.
Minimal Intervention for Stress-related mental disorders with Sick leave (MISS). Bakker et al.^16^	Stress-related mental disorders	Primary care generalist physicians received training to diagnose stress-related mental disorders and to detect symptoms of depression and anxiety. Then they were taught how to give information and to promote the patient’s understanding and how to emphasize the importance of the patient’s active role with regard to successful return to work. Subsequently, they practiced giving advice on the content of functional rehabilitation.	-	-
Music-based psychotherapeutic method focusing on workers’ problems and needs. Beck et al.^17^	Work-related stress	Music intervention based on the Bonny Method of Guided Imagery and Music.	-	-
Multidisciplinary intervention. Brendbekken et al.^18^	Chronic musculoskeletal pain	The multidisciplinary intervention included an assessment of work, family situation, lifestyle, coping strategies, and health problems. Furthermore, a novel educational tool was applied, the Interdisciplinary Structured Interview and a Visual Educational Tool (ISIVET) to establish an overall picture of the patient’s situation through visualization. The underlying hypothesis was that this design could introduce a new cognitive approach to cope with health problems.	-	-
Coordinated and tailored work rehabilitation. Bültmann et al.^19^	Musculoskeletal disorder	Identification of work disability and barriers for return to work. Coordinated, adapted worker-oriented rehabilitation plan.	-	The plan was sent to the worker’s general practitioner.
Multimodal cognitive behavioral treatment (MMCBT). Haldorsen et al.^20^	Musculoskeletal pain	Clear diagnosis, feedback of exams, information, relevant lessons, and physical training schemes. Cognitive coping strategies were discussed, and advice was given. The treatment was implemented partly as group activities and partly as individual training and therapy.	A visit to the workplace was performed to negotiate the labor changes required.	Telephone conferences with the company’s health service and/or construction supervisor on physical and psychological tensions in the workplace.
Occupational therapy. Hees et al.^21^	Major depression	Group and individual therapy sessions.	-	A meeting was held with the occupational therapist, the employer, and worker to discuss work difficulties.
Graded activity intervention. Hlobil et al.^22^	Low back pain	Graded activity intervention consisting of 60-minute exercise sessions twice a week until workers achieve full return to regular work.	-	-
Multidisciplinary intervention. Jensen et al.^23^	Low back pain	Physical exercise planning and medical treatment for pain.	-	The case manager and the worker developed a tailored rehabilitation plan for returning to work.
Integrated care. Lambeek et al.^24^	Chronic low back pain	Workplace intervention based on participatory ergonomics and a graded activity program, which is a time contingent program based on cognitive behavioral principles.	-	The clinical occupational physician, who was responsible for the planning and the coordination of the care and communication with the other healthcare professionals in the team, set a proposed date for full return to work in mutual agreement with the patient and the patient’s occupational physician. Communication between the team members consisted of phone calls, letters, coded email, and a conference call every three weeks discuss the progress of the patient regarding returning to work.
Cognitive-behavioral treatment complementary to a rheumatologic care program. Leon et al.^25^	Musculoskeletal disorder	Cognitive-behavioral treatment	-	-
Treatment as usual plus occupational therapy. Schene et al.^26^	Major depression	Group and individual therapeutic sessions.		Contact with worker’s occupational physicians on a work reintegration plan.
Workplace-based work hardening training. Cheng & Hung^27^	Rotator cuff disorder	Rehabilitation program.	-	The occupational coach communicated with the supervisor of the injured worker in the workplace to organize appropriate tasks as means of treatment that were appropriate to the current functional status of injured workers.
Psychiatric consultation for employees on sick leave for common mental disorders in the occupational health setting. Van der Feltz-Cornelis et al.^28^	Mental disorder	Psychiatric consultations.	-	-
Innovative activating intervention. Van der Klink et al.^29^	Adjustment disorders	Sessions with cognitive behavioral interventions, one session after return to work.	-	Contacts with the employer with regard to the intervention.
Participatory return-to-work program. Vermeulen et al.^30^	Musculoskeletal disorder	Identification of workplace problems with the worker and proposals of solutions.	Detection of workplace problems and proposals of solutions.	Detection of workplace problems and proposals of solutions together with the occupational specialist.
Collaborative care intervention focused on return to work. Vlasveld et al.^31^	Major depression	Individual therapy sessions, workplace intervention, and medications if needed.	Assessment of the workplace and adjustments.	Meeting between employers and workers to identify barriers for return to work and a plan to implement solutions.
Blended web-based intervention. Volker et al.^32^	Common mental disorder	Psychoeducation, a module aimed at cognitions with regard to return to work while having symptoms (based on cognitive behavioral therapy principles), a module aimed at increasing problem-solving skills with problem-solving treatment exercises, a module for pain and fatigue management and for reactivation, and a module for relapse prevention.	-	-
Cognitive behavioral therapy combined with meeting with the employer. de Weerd et al.^33^	Common mental disorder	Sessions with work-centered interventions.		A session with a convergence dialogue meeting with employers, workers, supervisors, and therapists, a dialogue session between workers and supervisors to identify and solve barriers for return to work.

The health problems described in the articles may be divided into: musculoskeletal disorders (10 articles, accounting for 52.6% of the total) and mental health disorders (nine articles, accounting for 47.4%). Among the articles on musculoskeletal disorders, five addressed non-specific musculoskeletal lesions (26.3% of the total); four, low back pain (21% of the total); and one, rotator cuff disorder (5.2% of the total). With regard to interventions that focused on mental health disorders, three assessed common mental disorders (15.7% of the total); three, depression (15.7%); two, stress (10.5%); and one, anguish (5.2%).

It was observed that all interventions (19) proposed actions with workers, such as rehabilitation programs, therapies, and return-to-work plans. In relation to actions in the workplace, only three interventions combined actions with workers and assessment of the workplace. Finally, actions with employers were considered in 10 interventions, in order to engage employers in workplace improvements and in the planning for worker’s return to work.

With regard to actions with workers in the interventions associated with musculoskeletal disorders, the interventions proposed by Brendbekken et al.,^18^ Bültmann et al.,^19^ Jensen et al.,^23^ and Lambeek et al.^24^ stand out, in which several healthcare professionals articulated themselves with proposals coordinated, adapted, worker-oriented work rehabilitation plans. In the interventions proposed by Haldorsen et al.^20^ and Leon et al.,^25^ mental health specialists discussed cognitive coping strategies and provided counseling for workers’ problems of pain and musculoskeletal disorder. In the interventions proposed by Hlobil et al.^22^ and Cheng & Hung,^27^ actions with workers consisted of rehabilitation programs and exercises according to patient’s needs and therapeutic goals. The intervention proposed by Arnetz et al.^15^ included a training program to adapt workers to tasks and to successive increase in workload and, finally, in the study by Vermeulen et al.,^30^ workplace problems were identified together with workers. As for actions focused on the workplace, the interventions proposed by Arnetz et al.,^15^ Haldorsen et al.,^20^ and Vermeulen et al.^30^ stand out, with assessment of the workplace and actions aimed at ergonomic improvements, detection of problems, and required work changes. Finally, with regard to actions with employers, the interventions conducted by Arnetz et al.,^15^ Haldorsen et al.,^20^ Jensen et al.,^23^ Lambeek et al.,^24^ Cheng & Hung,^27^ and Vermeulen et al.^30^ proposed contact with employers through meetings or telephone calls with the main purpose of identifying problems and barriers for return to work and consequent implementation of solutions.

In turn, among interventions linked to mental health problems and targeted to workers, the interventions proposed by Hees et al.^21^ and Schene et al.^26^ stand out, in which group therapy sessions are conducted and patients are taught to assessed the positive and negative factors of their own work situation. The interventions proposed by Van der Klink et al.^29^ and de Weerd et al.^33^ consisted of stimulating knowledge on problem-solving skills by patients and structuring their daily activities. In the intervention proposed by Bakker et al.,^16^ primary care practitioners were trained on the diagnosis of a stress-related mental disorder and subsequent counseling to workers. The music-based intervention proposed by Beck et al.^17^ applied a guided imagery and music intervention for workers’ problems and needs. The intervention proposed by Van der Feltz-Cornelis et al.^28^ applied an intervention based on supportive psychiatric consultations designed to deliver a diagnosis and treatment plan, including suggestions for return to work adapted to the specific workers’ needs. In their intervention, Vlasveld et al.^31^ proposed the application of sessions of brief structured psychological intervention, in addition to manually guided self-help and, finally, in the blended web-based intervention proposed by Volker et al.,^32^ an E-health module embedded in Collaborative Occupational health care (ECO) intervention including two parts: an eHealth module (Return@Work) and a decision aid via e-mail for the occupational physician. With regard to actions in the workplace, only the intervention proposed by Vlasveld et al.^31^ included the assessment of the workplace with adjustments. Finally, in relation to actions with employers, the interventions proposed by Hees et al.,^21^ Schene et al.,^26^ Van der Klink et al.,^29^ Vlasveld et al.,^31^ and de Weerd et al.^33^ included meetings with employers to identify barriers for return to work and improvement plans.

## DISCUSSION

With regard to the musculoskeletal diseases considered in the interventions, musculoskeletal disorders, such as non-specific musculoskeletal injuries and low back pain, were the most frequent. According to the Pan American Health Organization (PAHO), musculoskeletal disorders, such as low back pain, are one of the emerging diseases, as well as mental disorders, over the last 15 years.^1^

It bears highlighting that the literature shows the relationship between musculoskeletal diseases and working conditions. A study that aimed to related non-specific low back pain within the nursing work context revealed that, most of the 301 workers considered as unsatisfactory the items related to environmental temperature, inappropriate space, furniture, sanitary facilities, and rest. The authors conclude that changes in organizations and working conditions should occur in order to reduce the risks of workers’ illness.^34^

In the present systematic review, actions focused on the workplace proposed in the interventions by Arnetz et al.,^15^ Haldorsen et al.,^20^ and Vermeulen et al.^30^ corroborate with the need for changes in the work context. The importance of assessing workplace conditions and proposing ergonomic improvements and required work changes not only facilitates return to wok of workers on sick leave, but also prevents disease or its worsening.

With regard to actions targeted at workers on sick leave for musculoskeletal disorders, the interventions proposed by Arnetz et al.,^15^ Bültmann et al.,^19^ Hees et al.,^21^ Jensen et al.,^23^ Cheng & Hung,^27^ and Vlasveld et al.^31^ stand out, because they articulated the detection of work disability and barriers for return to work with coordinate, adapted, and work-oriented labor rehabilitation plans. Therefore, return-to-work intervention programs usually identified the barriers that could prevent workers from returning successfully to work and assessed their strengths and limitations. Then, a designated coordinator provides workers with tailored interventions to overcome these barriers.^14^

A systematic review that assessed the effectiveness of rehabilitation interventions in the workplace for workers with musculoskeletal low back pain clinical interventions with occupational interventions as therapeutic actions targeted at return to work in patients with low back pain from, as well as early return to work with modified work interventions, which coincides with the actions indicated in the interventions described in the present study.^35^

With regard to mental health disorders, interventions were designed for common mental disorders, depression, stress, and anguish; moreover, according to the WHO, depression and anxiety disorders are the leading cause of disability.^36^

Concerning actions of interventions focused on workers on sick leave due to mental disorders, in brief, they consisted of group and individual therapy sessions or psychiatric consultations focused on cognitive behavioral interventions aimed at problem resolution and identification of barriers for return to work. As for actions in the workplace, only the intervention proposed by Vlasveld et al.^31^ included the assessment of the workplace with adjustments.

A systematic review that aimed to evaluate the effectiveness of interventions designed to reduce work disability in employees with depressive disorder found that combining a clinical intervention focusing on the worker with a work-directed intervention probably reduces the number of days on sick leave.^37^

In relation to actions with employers, coordination return-to-work interventions depend on a good communication between the different concerned parties (i.e., workers, employers, supervisors, medical care providers, and insurers), as described in a systematic review that assessed the effects of return-to-work coordination programs for workers on sick leave or with a disability,^14^ an aspect that coincides with what is proposed in the present review, since return-to-work plans were jointly developed with workers and employers, and considering the workplace.^14,15,19,21,23,27,31^

Finally, this review has some limitations, such as the sample size of the selected studies, which would be improved so as to be more representative; moreover, other factors should be analyzed, such as the bias of each study and, thus, the quality of the evidence with regard to information and results delivered by each of them.

## CONCLUSIONS

In light of the proposed objective, it can be concluded that, in relation to musculoskeletal disorders, the interventions proposed the following actions with workers: identification of disability, kinesiology sessions, physical activity, and treatment for pain. For mental health diseases, the interventions proposed sessions with cognitive interventions, group and individual therapy sessions aimed at problem resolution and coping, in addition to identification of barriers for return to work and development of a coordinated, adapted, worker-oriented labor rehabilitation plan.

Concerning actions in the workplace, interventions proposed the assessment of ergonomic aspects and of physical and psychological stressful factors in the workplace.

Finally, with regard to employers, in-person meetings between the employer and the worker guided by the team were proposed, in order to identify difficulties in the workplace and in return to work, as well as the development of workplace improvement plans.

It is worth highlighting that the present study genera knowledge for the design of interventions and their validation for future studies.
